# Comparison of Objective Measures for Predicting Perceptual Balance and Visual Aesthetic Preference

**DOI:** 10.3389/fpsyg.2016.00335

**Published:** 2016-03-11

**Authors:** Ronald Hübner, Martin G. Fillinger

**Affiliations:** Department of Psychology, Universität KonstanzKonstanz, Germany

**Keywords:** visual aesthetics, perceptual balance, measures of balance, symmetry, homogeneity

## Abstract

The aesthetic appreciation of a picture largely depends on the perceptual balance of its elements. The underlying mental mechanisms of this relation, however, are still poorly understood. For investigating these mechanisms, objective measures of balance have been constructed, such as the Assessment of Preference for Balance (*APB)* score of Wilson and Chatterjee (2005). In the present study we examined the *APB* measure and compared it to an alternative measure (*DCM;* Deviation of the Center of “Mass”) that represents the center of perceptual “mass” in a picture and its deviation from the geometric center. Additionally, we applied measures of homogeneity and of mirror symmetry. In a first experiment participants had to rate the balance and symmetry of simple pictures, whereas in a second experiment different participants rated their preference (liking) for these pictures. In a third experiment participants rated the balance as well as the preference of new pictures. Altogether, the results show that *DCM* scores accounted better for balance ratings than *APB* scores, whereas the opposite held with respect to preference. Detailed analyses revealed that these results were due to the fact that aesthetic preference does not only depend on balance but also on homogeneity, and that the *APB* measure takes this feature into account.

## Introduction

The perceptual mechanisms involved in visual aesthetics and preference judgments have long been a matter of debate (for an overview see Palmer et al., 2013). Since the seminal work of Gustav Theodor Fechner (Fechner, 1871, 1876) one approach of corresponding experimental studies has been to find aesthetic primitives, i.e., relatively simple perceptual features that determine the attraction of a stimulus (Latto, 1995; Munar et al., 2014). A prominent candidate in this respect is *perceptual balance*, i.e., how well the elements in a picture are arranged. There is wide consensus among aestheticians that balance has a great effect on the appreciation of a picture (Poore, 1903; Arnheim, 1954). Nevertheless, the mechanisms of balance perception are still largely unknown. Whereas most researchers agree on a global level what perceptual balance is, they disagree on the details of how balance is determined. In the present article we consider currently applied measures and examine how they are related to subjective balance, symmetry, and aesthetic preference.

Similar to early ideas of the artist and writer Henry Poore (1859-1940, Poore, 1903), and, as revealed by McManus et al. (2011), based on the work of Denman Ross (1853-1935, Ross, 1907), the Gestalt psychologist Rudolf Arnheim (1904-2007) hypothesized in his book *Art and Visual Perception* (Arnheim, 1954) that each rectangular frame has a hidden structure or field of invisible forces (analog to a magnetic field in physics). The center of the frame has the strongest attraction, followed by the corners, the two main axes, and the diagonals. If an element is placed in the frame, then it is pulled by all the forces of the hidden structure, which produces an inner tension or *psychological force* of that element in relation to the square. For instance, if a single element is placed at the center, then all forces compensate each other and the picture is perfectly balanced. In contrast, if the element is placed off-center, then there is a pull toward the center, which results in imbalance. The situation is obviously more complex if several elements are placed in a frame. In this case each element has a relative perceptual weight resulting not only from the hidden forces of the frame, but also from forces originating from the other elements. A picture is perceived as balanced if these weights compensate each other. Furthermore, as proponent of Gestalt psychology, Arnheim (1954) also assumed that perceptual grouping (by similarity of form, color, etc.) modulates the forces between the elements.

An alternative characterization of perceptual balance is to consider the subjective equilibrium of a picture. According to Arnheim (1954), every visual pattern has a center of perceptual “mass,” which depends on the perceptual weight of the elements. If this center coincides with the geometric center of the frame, then the picture is balanced. It is assumed that the perceptual weight of an element increases proportionally to the element's distance from the center of “mass” (lever principle in physics). However, the weight also depends on factors such as element size (larger elements are perceptually heavier than smaller ones), color (e.g., red is perceptually heavier than blue), and regularity (regular shapes are perceptually heavier than irregular ones). Arnheim conceded that most of these factors have to be verified, which is still valid today.

Some of Arnheim's (1954) main assumptions have already been tested. McManus et al. (1985), for instance, presented reproductions of art work as well as plain stimuli, and had their participants to place a fulcrum beneath each picture so that it looked balanced (horizontally). For the reproductions of art work they found that the adjusted position of the fulcrum varied considerably, suggesting that art work is not generally well balanced. Moreover, when participants had to locate the perceptual center for unchanged pictures and for pictures where a portion was removed, the locations were rather similar. From these results McManus et al. (1985) concluded that the balance of a picture depends more “…upon a global integration of the picture as a whole, than of any individual element of it” (p. 314f).

Even for their plain stimuli McManus et al. (1985) found no simple relation. Whereas element position was crucial for positioning the fulcrum, size and color of the elements were less important. Furthermore, although the distance of an element from the frame's geometric center and its size led to a larger shift of the fulcrum, these two factors were not correctly integrated for the judgment of balance.

In a later study, Locher et al. (1996) used reproductions of twentieth-century art paintings and a manipulated less-balanced version of each. Art experts and non-experts had to rate the balance of each picture and to determine the (two-dimensional) center of perceptual “mass.” As a result, both groups moved the center for the disrupted version, but only the experts judged this version as less balanced. Locher et al. (1996) concluded that the center of perceptual “mass” and the overall judgment of balance are not as close as thought.

Because these results do hardly support Arnheim's theory, Cupchik (2007) speculated that the terms of the theory were only meant metaphorically. McManus et al. (2011), however, believed that Arnheim wanted his theory to be taken literally, i.e., in a physical sense. To test their conjecture, they even went a step further and, instead of asking participants to indicate the fulcrum of pictures, calculated the center of “mass” by assuming that the “mass” of each pixel in a (gray-level) picture corresponds to the inverse of the pixel's gray level. They then examined whether the center was closer to an axis for art photographs than for control images, which was indeed the case.

Other tests, however, failed. For instance, in one experiment where McManus et al. (2011) presented simple pictures with only two discs but of a different gray level, performance was incompatible with a physical interpretation of balance. In view of these results, McManus et al. (2011) also came to the conclusion that the terms in Arnheim's theory cannot be taken literally.

**Figure 1:**
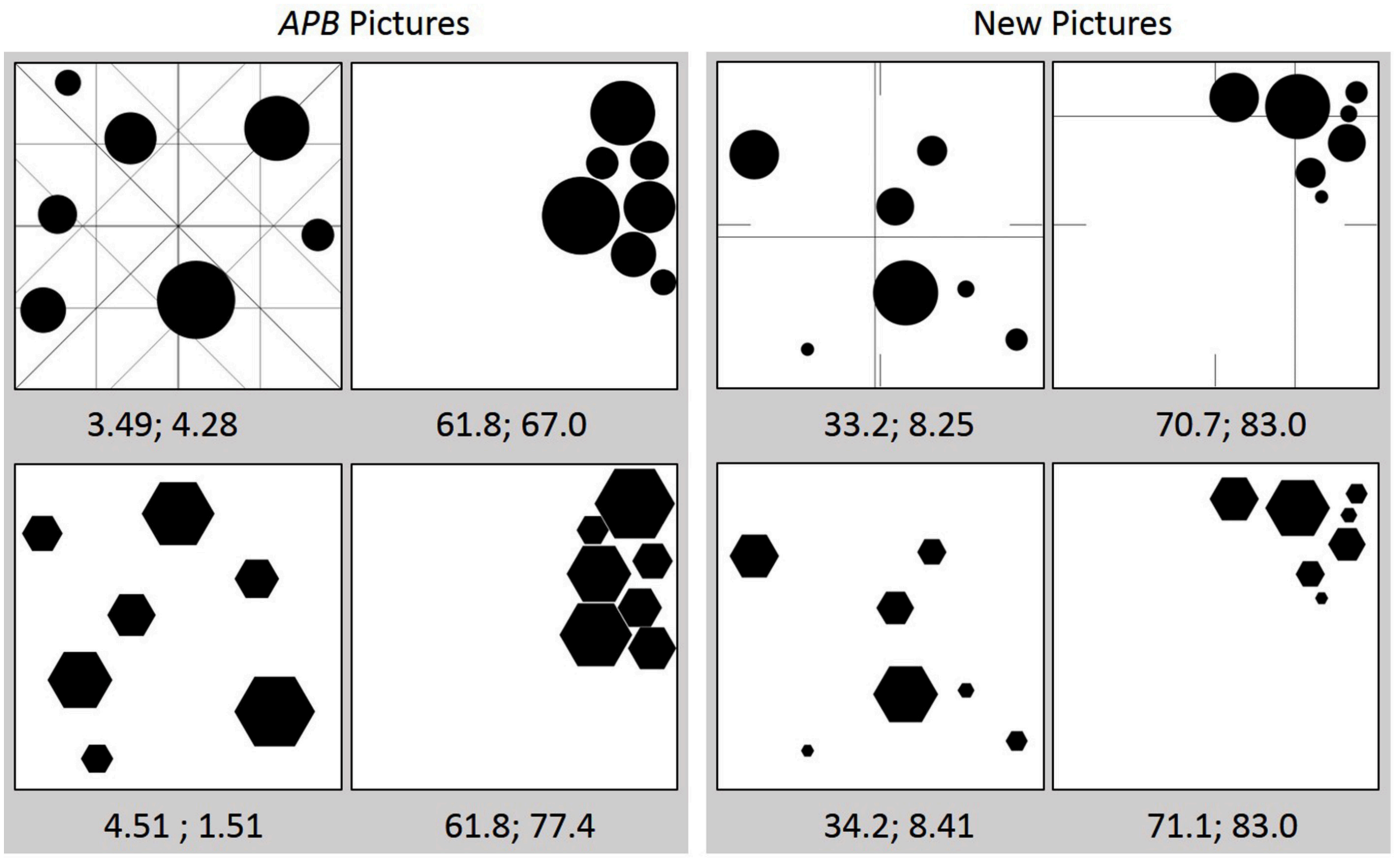
**Example stimuli used in the experiments. Left panel:** Pictures from the APB (Wilson and Chatterjee, 2005). The first and second number below each picture indicates the *APB* (computed with our algorithm) and *DCM* score, respectively. Note: the lower the value the higher the balance. In the top left figure additionally the different axes are shown for the demonstration of how the *APB* score is computed (see text for details). **Right panel**: Examples of the new stimuli. The intersections of the long lines in each picture indicate the respective center of “mass” (see text for details). The short lines imply the corresponding geometric center.

The considered studies suggest that perceptual balance is a complex feature of pictures that depends on several factors, whose details are still largely unknown. However, the studies also demonstrate that computing objective measures for predicting subjective balance and preference is a promising approach for investigating these matters. As we have seen, McManus et al.'s (2011) physical interpretation of perceptual “mass” was not successful in this respect. However, there are other measures. Wilson and Chatterjee (2005), for instance, developed a test for the *Assessment of Preference for Balance* (APB). In connection with this test they introduced a measure, which we will call “*APB*” that highly correlated with perceptual balance and preference (liking), at least for simple pictures such as shown in Figure 1.

That the applicability of the *APB* measure might indeed be restricted to simple pictures is suggested by results of Gershoni and Hochstein (2011), who found only small correlations between this measure and ratings for Japanese calligraphy. Nevertheless, even if the measure predicts only preference between simple stimuli, it could be the starting point for the development of more sophisticated measures that also apply to complex pictures. Unfortunately, it is not even sure that the *APB* measure is valid for simple images. For instance, in a study by Silvia and Barona (2009), who used a subset of Wilson and Chatterjee's (2005) stimuli, no substantial correlation between *APB* scores and liking was observed.

One aim of the present study was to replicate Wilson and Chatterjee's (2005) results by applying complete sets of their original images. Furthermore, because the *APB* measure is the average of eight components, it was possible to use multiple regression analyses to examine the extent to which the components are related to perceptual balance and aesthetic preference. Such analyses have not been done before. A second aim of our study was to compare the *APB* measure to three other objective measures that have also been proposed for measuring balance: a measure of balance that is based on the physical interpretation of perceptual “mass,” a measure of mirror symmetry, and a measure of heterogeneity. Finally, we wanted to examine to what extent the results can be generalized. Therefore, we also applied new sets of stimuli.

For replicating Wilson and Chatterjee's (2005) results and for comparing the *APB* measure with alternative measures, we conducted two experiments. In the first one we collected balance and symmetry ratings for pictures from the APB and examined how well the different measures can account for the judgments. In the second experiment different participants rated the same pictures with respect to aesthetic preference (liking). The ratings were then correlated with the judgments from Experiment 1, and with the different measures. As we will show, our results were similar to those of Wilson and Chatterjee's (2005). However, some of the alternative measures were also highly correlated with balance or preference ratings. A third experiment, where participants had to rate the balance as well as the liking of new stimuli, revealed that the specific selection of stimuli has some effects on the results. Before we report our results in detail, however, we introduce the applied measures.

### Assessment of preference for balance (APB)

Wilson and Chatterjee's (2005) test for the APB consists of images containing seven black elements of varying sizes that are scattered on a white quadratic background (750 × 750 pixels). There are 65 images with circles, hexagons, or squares, respectively. All elements within each image have the same shape (for examples see Figure 1). To also have an objective measure of balance for each picture, they created a specific score, defined by the mean of eight partial measures that are more or less related to symmetry. Relying on symmetry seems to be reasonable, because this feature is strongly related to balance and preference. Mirror symmetry, for instance, is the simplest form of balance. Accordingly, symmetric pattern can not only be processed and remembered more easily than asymmetric ones (Garner and Clement, 1963), they are also judged as more “beautiful” (Jacobsen and Höfel, 2002). On the other hand, balance can be understood as a more complex form of symmetry (Locher and Nodine, 1989).

For obtaining the *APB* score, two symmetry measures are computed around the vertical and the horizontal axes, and around the two diagonal axes, respectively. Assume that a picture is divided along the horizontal dimension into four vertical, equally sized rectangles (see upper left picture in Figure 1), denoted by A_1_, A_2_, A_3_, and A_4_, from left to right, respectively. If *f* denotes a function that counts the number of black pixels in a given area, then the number *N* of all such pixels in a picture is *f* (A_1_) + *f* (A_2_) + *f* (A_3_) + *f* (A_4_). The first partial symmetry measure for the horizontal dimension (around the vertical axis) is defined by *h* = (|[*f* (A_1_) + *f* (A_2_)] – [*f* (A_3_) + *f* (A_4_)]|/*N*)·100, i.e., the absolute difference between the number of black pixels in the left half and that in the right half of the picture in percent. The second measure for this dimension reflects the so-called horizontal *inner-outer relation* and is defined by *h*_*io*_ = (|[*f* (A_1_) + *f* (A_4_)] − [*f* (A_2_) + *f* (A_3_)]|/*N*)·100. Analogous partial measures are computed for each of the remaining three axes (the corresponding divisions of the picture area are shown in the upper left picture in Figure 1). The corresponding measures for the vertical dimension are denoted by *v* and *v*_*io*_, those for the main diagonal (top left to bottom right) by *md* and *md*_*io*_, and those for the anti-diagonal by *ad* and *ad*_*io*_. Finally, the mean of the eight partial measures defines the *APB* score. Note that a low score (percentage) means high balance, whereas a high score reflects poor balance.

### Deviation of the center of “mass” (DCM)

Because the *APB* score is only loosely related to physics, we also applied a measure that is more strongly related to a physical interpretation of balance in the sense of Arnheim (1954). For this objective we computed a measure that represents the *deviation of the center of “mass”* (*DCM*) from the picture's geometrical center. Assume two elements with visual “masses” *m*_1_ and *m*_2_ respectively, arranged on a beam. A point located between these objects at distance of *d*_1_ and *d*_2_, respectively, is the center of “mass” (balance point, fulcrum) if *m*_1_*d*_1_ = *m*_2_*d*_2_. A practical way to calculate the center is to calculate the distances *r*_1_ and *r*_2_ of the “masses” from an arbitrary reference point (see McManus et al., 2011). The balance center is then located at distance *r* = (*m*_1_*r*_1_ + *m*_2_*r*_2_)/(*m*_1_ + *m*_2_).

For the black-and-white pictures used in this study, we assumed that the “mass” of a black pixel is one, whereas that of a white pixel is zero. If we chose position *x* = 0 as reference point, then the center of “mass” *b*_*x*_ on the horizontal dimension is located at position:

$$\begin{array}{l} b_{x}=\frac{\sum_{i=1}^{w}m_{i}r_{i}}{\sum_{i=1}^{w}m_{i}}, \end{array}$$

where *w* is the picture width, and *m*_*i*_ the number of black pixels in column *i*. The center for the vertical dimension is calculated analogously. In Figure 1, the line intersections in the two upper right pictures indicate the respective locations of the center of “mass.” The geometric centers are implied by the short lines.

In the present study we used the normalized location $b_{x}^{\prime }=b_{x}$/*w*, which can vary from zero to one. For these coordinates the geometrical center is at 0.5, and the horizontal distance to the center of “mass” is *d*_*x*_ = 0.5-*b*′_*x*_. An analog distance *d*_*y*_ was calculated for the vertical dimension. The *DCM* measure of balance is then defined by the Euclidean distance of the two-dimensional center of visual “mass” to the geometrical center of the image. Specifically, we used the relative deviation in percent:

$$\begin{array}{l} DCM=\left(\frac{\sqrt{d_{x}^{2}+d_{y}^{2}}}{0.5}\right)100. \end{array}$$

### Mirror symmetry (MS)

As shown, the *APB* score is the mean of different measures most of which are based on the symmetry around some axis of the picture. Symmetry, however, is reflected only coarsely by these measures. Therefore, we also considered a measure of mirror symmetry (*MS*) that is defined by the mean of mirror-symmetry measures around different axes. The partial score for a given axis was computed by a formula suggested by Bauerly and Liu (2006). Assume that the vertical axis is the axis of reflection and that *m* and *w* denote the height and width of the image in pixels, respectively. The required number of comparisons *n* for each row is *w*/2, if *w* is even and (*w*-1)/2, if *w* odd. Assume further a binary variable *X*_*ij*_ that is 1 if there is a match between pixels and 0, otherwise. Finally, there is a factor that reduces the weight of the match the farther away from the axis of reflection it is. The symmetry *s* for the vertical axis is then:

$$\begin{array}{l} s=\frac{2}{3mn}\overset{m}{\underset{i=1}{\sum }}\overset{n}{\underset{j=1}{\sum }}X_{ij}\left(1+\frac{j-1}{n-1}\right). \end{array}$$

Analogous measures were computed for the horizontal axis and for each of the two diagonals. At the end, the four measures were multiplied by 100 and averaged. The resulting *MS* score is the mean symmetry in percent. The higher the value the more symmetric the picture.

### Homogeneity (HG)

If we consider the pictures of the APB (for examples see the left panel in Figure 1), then it is obvious that balance is confounded to some extent with *homogeneity*. For many pictures it holds that, the less scattered the elements in the picture, the less balanced the picture. To investigate this relation in detail, we also wanted to include a measure of homogeneity. A measure that reflects this feature and that has widely been applied, among others for evaluating the design of user interfaces (e.g., Ngo et al., 2002), is information entropy (Shannon, 1948). Assume that we divide the picture area into *M* equally sized regions (bins). The entropy *E* is then defined by:

$$\begin{array}{l} E=-\overset{M}{\underset{i=1}{\sum }}p_{i}\ln\;p_{i}, \end{array}$$

Where *p*_*i*_ is the probability of black pixels in bin *i*, which is usually estimated by the corresponding relative frequency. For a given number of bins, the maximum entropy is reached if all bins contain the same number of black pixels. The value of this maximum is ln(*M*). Thus, a proper score of picture homogeneity can be obtained by calculating the relative entropy:

$$\begin{array}{l} E_{r}=\frac{E}{\ln\;M}. \end{array}$$

For the present study we computed separate values *E*_*rx*_ and *E*_*ry*_ for the horizontal and vertical dimension, respectively. For each dimension we divided the picture into 10 bins along the corresponding axis. The score *HG*, which reflects homogeneity in percentage, is then:

$$\begin{array}{l} HG=\left(\frac{E_{rx}+E_{rx}}{2}\right)100. \end{array}$$

## Experiment 1

In our first experiment we collected balance and symmetry ratings for two sets of pictures (constructed from circles or from hexagons) taken from the APB (Wilson and Chatterjee, 2005) and examined to what extent these ratings correlate with the objective measures of balance, symmetry, and homogeneity.

### Method

Participants were 18 students from the University of Konstanz. They were recruited via an online system (ORSEE, Greiner, 2015) for participating in the experiment. The data of two participants were excluded from data analysis, because one of them produced many extreme values (0 and 100), and the other misunderstood the rating scales. The remaining 16 participants (3 males) had an average age of 23 years (*SD* = 1.77). All had normal or corrected-to-normal vision and were paid 8 € for their participation. The experiment was performed in accordance with the ethical standards laid down in the 1964 Declaration of Helsinki and its later amendments. In agreement with the ethics and safety guidelines at the Universität Konstanz, we obtained a verbal informed consent statement from all individuals prior to their participation in the study. Potential participants were informed of their right to abstain from participation in the study or to withdraw consent to participate at any time without reprisal.

#### Apparatus and stimuli

The stimuli were presented on a 19″ LCD-monitor with a resolution of 1280 × 1024 pixels. A personal computer (PC) served for controlling stimulus presentation and response registration. As stimuli served all 65 pictures with circles and all 65 pictures with hexagons from Wilson and Chatterjee's (2005) APB. The pictures consisted of seven elements, which had the same shape, but varied in size. The *APB* score for each stimulus was calculated by our own algorithm, which produced values quite close (*r* > 0.999) to those provided by Wilson and Chatterjee (2005). Across pictures, the *APB* scores ranged from 3.49 to 65.9 (*M* = 35.3, *SD* = 17.4). *DCM* scores ranged from 1.51 to 79.5 (*M* = 35.4, *SD* = 25.1), homogeneity (entropy) from 64.1 to 94.8 (*M* = 82.9, *SD* = 7.71), and mirror symmetry from 1.13 to 10.9 (*M* = 3.91, *SD* = 1.65). The stimuli were presented at the center of the monitor on a black background. Each picture had an extension of 750 × 750 pixels, which approximately corresponded to a visual angle of 21° horizontally and vertically.

#### Procedure

After the participants had read the instruction and considered 6 example stimuli (3 with circles and 3 with hexagons), which were not used for the main task, they rated each picture with respect to balance and symmetry. Instead of a 1-to-5 rating scale, as in Wilson and Chatterjee (2005), we applied a continuous scale (1-to-100 slider bar) to reduce information loss (cf. Treiblmaier and Filzmoser, 2009). The scale went from “not balanced” to “balanced” for the balance rating, and from “not symmetrical” to “symmetrical” for the symmetry rating. The participants saw a horizontal slider located below the stimulus and had to move a computer mouse to adjust the position of the slider that corresponds to their subjective estimation of balance or of symmetry, respectively. The corresponding numeric value (not visible for the participants) of the chosen position was then entered by clicking the left mouse button. There was no time limit. Immediately after the value was entered, the next stimulus was displayed.

Balance and symmetry were assessed in alternating blocks of 130 trials. Half of the participants started with rating balance, the other half with rating symmetry. There were two blocks for balance and symmetry rating, respectively. The 130 pictures (65 with circles and 65 with hexagons) were randomized within each block. The experiment lasted approximately 50 min.

### Results

#### Balance ratings

The mean balance ratings ranged from 16.2 to 79.3 (*M* = 43.2, *SD* = 15.3). They were subjected to a one-way within-participant ANOVA with factor *stimulus type* (circles, or hexagons). There was no significant difference (circles: 45.5, hexagons: 41.0) between the stimulus types, *F*_(1, 15)_ = 2.88, *p* = 0.113, $\eta_{p}^{2}$ = 0.159.

##### APB

The mean *APB* scores for pictures with circles and with hexagons were 34.9 and 35.7, respectively. In a first step we computed for each participant the correlation between the balance ratings and the scores across the 65 pictures with circles and across the 65 pictures with hexagons. For the pictures with circles the correlations ranged from −0.075 to −0.860, and for those with hexagons from −0.132 to −0.855. There was only one participant with non-significant (*p* > 0.05) correlations for both stimulus types. Three participants had a non-significant correlation for one of the stimulus types. The mean correlations are listed in Table 1.

**Table 1:** **Means (across participants) of the individual correlations (across pictures) between the balance and symmetry ratings and the different scores in Experiment 1**.

	***APB***	***DCM***	***MS***	***HG***
Balance circles	−0.450 (0.222)	−0.479 (0.243)	0.269 (0.152)	0.378 (0.208)
Balance hexagons	−0.484 (0.207)	−0.493 (0.212)	0.252 (0.087)	0.444 (0.201)
Symmetry circles	−0.670 (0.231)	−0.677 (0.239)	0.234 (0.141)	0.592 (0.197)
Symmetry hexagons	−0.712 (0.200)	−0.723 (0.120)	0.247 (0.079)	0.661 (0.158)

*The values in parenthesis are the standard deviations*.

*APB, Assessment of preference for balance; DCM, Deviation of the center of “mass”; MS, Mirror symmetry; HG, Homogeneity*.

Next, we computed the mean balance ratings across participants for each picture and correlated the obtained values across all 130 pictures with the different scores. The correlations and corresponding *R*^2^-values are shown in Table 2.

**Table 2:** **Correlations between the mean ratings and the different scores across both stimulus types in Experiment 1**.

	**Balance**	**Symmetry**	***APB***	***DCM***	***MS***	***HG***
Balance	–	0.929^***^	−0.784^***^	−0.822^***^	0.418^***^	0.707^***^
Symmetry	0.864	–	−0.909^***^	−0.926^***^	0.314^***^	0.833^***^
*APB*	0.615	0.826^***^	–	0.866^***^	−0.177^***^	−0.852^***^
*DCM*	0.675	0.857^***^	0.751^***^	–	−0.389^***^	−0.798^***^
*MS*	0.175	0.099^***^	0.031^***^	0.151^***^	–	0.057^***^
*HG*	0.500	0.694^***^	0.725^***^	0.637^***^	0.003	–

*The values below the diagonal are the corresponding R^2^-values*.

*** *p < 0.001. APB, Assessment of preference for balance; DCM, Deviation of the center of “mass”; MS, Mirror symmetry; HG, Homogeneity*.

As mentioned, the *APB* scores are the mean of eight different measures. This implies that each component has the same weight. To examine whether this is appropriate, we also computed a multiple linear regression for each of the two stimulus types and for both types together. The results are shown in Table 3. If we consider the regression across both stimulus types, then we see that *R*^2^ increased for the *APB* score (Table 2) from 0.615 to 0.751, which demonstrates that different weights for the components can improve the predictive power of the score. The obtained individual coefficients indicate that the horizontal component (symmetry over the vertical axis) has by far the largest weight. In contrast, the inner-outer components hardly explained variance.

**Table 3:** **Regressions of balance ratings on the components of the *APB* scores in Experiment 1**.

	**Circles (*****R***^**2**^ = 0.747**)** ***F***_**(8, 56)**_ = 20.6, ***p*** < 0.001 **Intercept** = **62.1**		**Hexagons (*****R***^**2**^ = 0.784**)** ***F***_**(8, 56)**_ = 25.3, ***p*** < 0.001 **Intercept** = **63.5**		**Both (*****R***^**2**^ = 0.751**)** ***F***_**(8, 121)**_ = 45.5, ***p*** < 0.001 **Intercept** = **63.1**	
	**β**	***P(>|t|)***	**β**	***P(>|t|)***	**β**	***P(>|t|)***
*h*	−0.218	0.000^***^	−0.259	0.000^***^	−0.229	0.000^***^
*v*	−0.039	0.323	−0.1037	0.040^*^	−0.076	0.014^*^
*ad*	−0.066	0.195	−0.077	0.137	−0.082	0.019^*^
*md*	−0.096	0.026^*^	−0.064	0.204	−0.087	0.007^**^
*h_*io*_*	0.015	0.769	0.039	0.443	0.032	0.348
*v_*io*_*	−0.015	0.737	−0.098	0.051	−0.057	0.083
*ad_*io*_*	0.041	0.384	−0.008	0.874	0.011	0.736
*md_*io*_*	−0.070	0.165	0.003	0.949	−0.030	0.364

* p < 0.05;

** p < 0.01;

*** *p < 0.001*.

##### DCM

The mean *DCM* scores for circles and hexagons were 33.3 and 37.3, respectively. Correlations of the *DCM* scores with the balance ratings for individual participants ranged from −0.008 to −0.786 for circles and from −0.153 to −0.861 for hexagons. The mean correlations are listed in Table 1. As can be seen, the correlations for the *DCM* scores were somewhat higher than those for the *APB* scores. However, a comparison across both stimulus types revealed no significant difference (−0.467 vs. −0.486), *F*_(1, 15)_ = 2.11, *p* = 0.167, $\eta_{p}^{2}=0.123$. If we consider the mean balance ratings (see Table 2), then their correlation with the *DCM* scores was also numerically larger than that with the *APB* scores.

Because the *DCM* score represents the Euclidian distance to the image center, it is interesting to examine whether a linear combination of the horizontal and the vertical distance would have been a better measure. Therefore, we computed a multiple linear regression with these two components. As a result, there was a strong contribution of the horizontal deviation (see Table 4). However, *R*^2^ was smaller than the corresponding value for the *DCM* score (0.605 vs. 0.675). Thus, the Euclidian distance of the center of “mass” from the image center is a better measure than the linear combination of the horizontal and the vertical deviation.

**Table 4:** **Regressions of balance ratings on the components of the *DCM* score in Experiment 1**.

	**Circles (*****R***^**2**^ = 0.629**)** ***F***_**(2, 62)**_ = 55.0, ***p*** < 0.001 **Intercept** = **63.9**		**Hexagons (*****R***^**2**^ = 0.597**)** ***F***_**(2, 62)**_ = 45.8, ***p*** < 0.001 **Intercept** = **53.7**		**Both (*****R***^**2**^ = 0.605**)** ***F***_**(2, 127)**_ = 97.1, ***p*** < 0.001 **Intercept** = **57.0**	
	**β**	***P(>|t|)***	**β**	***P(>|t|)***	**β**	***P(>|t|)***
Horizontal	−52.1	0.000^***^	−60.3	0.000^***^	−56.8	0.000^***^
Vertical	−6.72	0.397	3.67	0.655	−0.969	0.867

*** *p < 0.001*.

##### MS

The mean scores of mirror symmetry for circles and hexagons were 3.21 and 3.36, respectively. Correlations between the symmetry ratings and the *MS* scores for the individual participants varied between 0.058 and 0.333 for circles and between −0.043 and 0.317 for hexagons. The mean values, which are shown in Table 1, were relatively small, as was the correlation between the mean balance ratings and the *MS* scores (see Table 2). A regression of the balance ratings on the four components of the *MS* score improved *R*^2^ only slightly from 0.175 to 0.196 (see Table 5). Although the diagonals significantly accounted for the balance ratings, the horizontal dimension (vertical axis of reflection) had the strongest effect.

**Table 5:** **Regressions of balance ratings on the components of the *MS* measure in Experiment 1**.

**Axis of reflection**	**Circles (*****R***^**2**^ = 0.281**)** ***F***_**(4, 60)**_ = 5.86, ***p*** < 0.001 **Intercept** = **32.6**		**Hexagons (*****R***^**2**^ = 0.189**)** ***F***_**(4, 60)**_ = 3.49, ***p*** < 0.05 **Intercept** = **31.8**		**Both (*****R***^**2**^ = 0.196**)** ***F***_**(4, 125)**_ = 7.61, ***p*** < 0.001 **Intercept** = **29.0**	
	**β**	***P(>|t|)***	**β**	***P(>|t|)***	**β**	***P(>|t|)***
Horizontal	0.078	0.882	1.017	0.134	0.570	0.179
Vertical	2.023	0.001^*^	1.595	0.007^**^	1.59	0.000^***^
Maj. diag.	0.472	0.322	1.122	0.073	0.851	0.028^*^
Min. diag.	0.972	0.049^*^	0.364	0.544	0.720	0.062

* p < 0.05;

** p < 0.01;

*** *p < 0.001*.

##### HG

The mean *HG* scores for circles and hexagons were 83.8 and 82.0, respectively. Correlations of the *HG* scores with the balance rating ranged for individual participants from 0.037 to 0.801 for circles and from −0.008 to 0.770 for hexagons. Mean correlations are shown in Table 1. The correlations for the *HG* measure are smaller than those for the *APB* and *DCM* scores. Such a pattern also occurred for the correlations with mean balance ratings (see Table 2). A statistical test revealed that the mean correlation for the *HG* scores was significantly smaller than that for the *APB* scores (−0.411 vs. −0.467), *F*_(1, 15)_ = 25.6, *p* > 0.001, $\eta_{p}^{2}=0.631$.

To examine how the two dimensions of the *HG* measure are related to the balance ratings, we computed a multiple linear regression with the corresponding two components. It revealed that homogeneity along the vertical dimension was as important as that along the horizontal dimension. Accordingly, the regression did not increase *R*^2^.

#### Symmetry ratings

The symmetry ratings differed significantly between the two stimulus types, $F_{\left(1,\;15\right)}=14.0,p<0.01,\eta_{p}^{2}=0.484$, indicating that the pictures with circles were perceived as more symmetric than those with hexagons (47.7 vs. 42.5). The mean correlations between the symmetry ratings and the different measures are shown in Table 1. Obviously, the symmetry ratings were rather weakly correlated with the *MS* scores. Six participants had at least one non-significant correlation (*p* > 0.05). Interestingly, the *APB* scores correlated higher with the symmetry ratings than with the balance ratings (−0.691 vs. −0.467), $F_{\left(1,\;15\right)}=12.9,p<0.01,\eta_{p}^{2}=0.462$, which was also the case for the *DCM* scores (−0.670 vs. −0.486), $F_{\left(1,\;15\right)}=12.8,p<0.01,\eta_{p}^{2}=0.461$, and for the *HG* scores (0.627 vs. 0.411), $F_{\left(1,\;15\right)}=13.9,p<0.01,\eta_{p}^{2}=0.481$.

The mean correlations were also somewhat larger for the *DCM* scores than for the *APB* scores (−0.691 vs. −0.700), which, however, was not significant, *F*_(1, 15)_ = 0.610, *p* = 0.447, $\eta_{p}^{2}=0.039$. A similar pattern of correlations occurred for the mean scores. Table 2 shows that the symmetry ratings correlated highly with the balance ratings (shared variance was 86%). In view of this correspondence we did not further analyze the symmetry ratings and their relation with the different measures.

#### Discussion

Our results show that the mean ratings for balance and symmetry were highly correlated, which suggests that it was difficult for the participants to operationalize the two concepts differently. That the two ratings were nevertheless not identical is indicated by the fact that the symmetry ratings were significantly higher for pictures with circles than for those with hexagons, which was not the case for the balance ratings. Moreover, the *APB, DCM*, and *HG* scores correlated higher with the symmetry ratings than with the balance ratings.

With respect to the *APB* scores, we replicated the result of Wilson and Chatterjee (2005). The mean scores correlated highly with the mean ratings of balance. However, it also became clear that the individual correlations were much smaller and varied considerably across participants. Furthermore, a regression analysis of the mean balance ratings on the components of the *APB* scores revealed that the components accounted differently for the balance ratings. The horizontal dimension had the largest effect, followed by the vertical one. Whereas the diagonal components also contributed to a small but significant extent, the inner-outer components had a negligible effect. In all, a differential weighting of the individual components increased the percentage of explained variance, compared to the original score with equal weights (averaging).

The *DCM* scores correlated surprisingly high with the ratings. The correlations were numerically even higher than those for the *APB* scores, which shows that it was actually not necessary to invent a new score for measuring balance. The *HG* scores also correlated substantially with subjective balance and symmetry, although not as high as the *APB* and the *DCM* scores. Interestingly, in contrast to the other measures, the vertical dimension was similarly importance for this correlation than the horizontal one. The *MS* scores had the weakest relation to the ratings, suggesting that perception does not take mirror symmetry into account, at least not for the current type of pictures.

Taken together, the results show that objective measures can be constructed that reflect perceptual balance (and symmetry), at least for the relatively simple pictures used here. A straightforward method is simply to compute how much the center of “mass” deviates from the geometric center of the picture. The larger the deviation the less balanced the picture. Another method would be to compute *APB* scores. However, although this measure also correlated highly with the ratings, a closer look at the pictures reveals an inconsistency. Table 6 includes three pictures from the APB whose *APB* scores increase from left to right. Obviously, Pictures #45 is less balanced than Picture #27. However, it is hard to believe that picture #46 shall be less balanced than Picture #45. That this is inconsistent to one's impression is also confirmed by our balance ratings. Picture #46 received a rating that was even higher than that of Picture #27. In contrast to the *APB* score, the *DCM* measure reflects this order. This strongly favors of the *DCM* score as measure for perceptual balance.

**Table 6:** **Example pictures from the APB and corresponding ratings and objective scores**.

	** 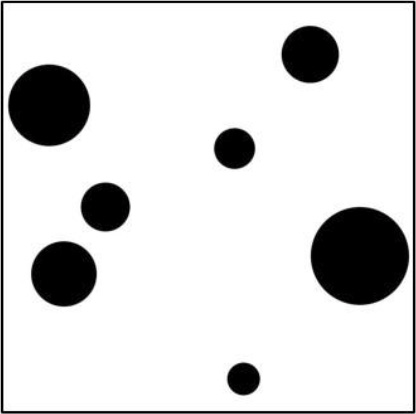 **	** 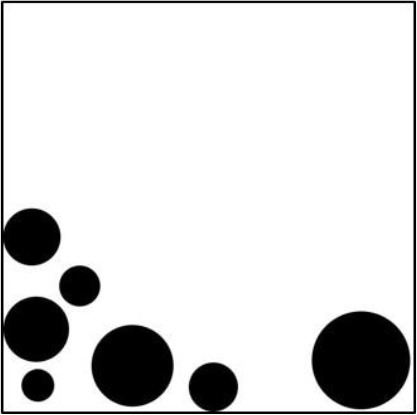 **	** 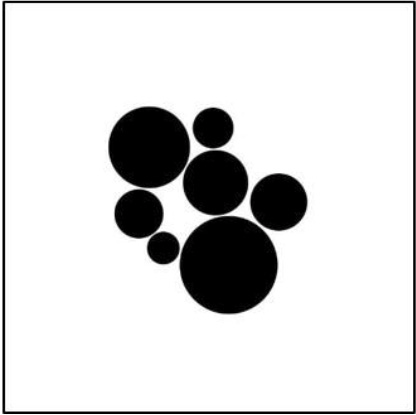 **
**Nr**.	**#27**	**#45**	**#46**
Balance (Experiment 1)	54	36	61
Symmetry (Experiment 1)	76	32	52
Liking (Experiment 2)	66	39	51
*APB*	24	46	47
*DCM*	12	67	2.7
*MS*	2.2	3.7	11
*HG*	91	79	72

An analysis of the components of the *APB* measure revealed that the reason for this inconsistency are the inner-outer components, which represent the difference in black pixels between the inner and the outer areas. Consequently, if elements are present only in the center, as in Picture #46, then these components have a high value, indicating unbalance, which however, does not reflect subjective balance. If we consider the different measures in Table 6, then it is obvious that the inner-outer components correspond to homogeneity. Indeed, homogeneity is highest for Picture #27. Thus, the *APB* measure is not very well suited for representing balance.

## Experiment 2

In our second experiment we wanted to collect preference ratings for the pictures shown in the first experiment. To avoid any influence from a second task, the participants had merely to indicate how much they liked each picture. The main goal was to examine to what extent the different ratings from Experiment 1 and the introduced measures can account for preference judgments.

In this context we also wanted to replicate the results of Wilson and Chatterjee (2005), who found a high correlation between their *APB* score and liking. In a subsequent study, Silvia and Barona (2009) could not replicate this result. However, their main goal was to test the hypothesis that pictures with curved elements are preferred to those with angular elements (Bar and Neta, 2006). Therefore, they applied only a selection of 9 pictures with circles and one of 9 pictures with hexagons from the APB to construct three different levels of balance. Whereas pictures with circles were indeed preferred to those with hexagons, the correlation between *APB* score and liking was rather low.

### Method

Twenty-one students (5 male) from the University of Konstanz with an average age of 24 years (*SD* = 3.11) participated in the experiment. They were recruited in the same way as in Experiment 1, and no one had participated in Experiment 1. All had normal or corrected-to-normal vision and were paid with 4 € for their participation. The experiment was performed under the same ethical standards as the previous experiment.

Stimuli and apparatus were the same as in Experiment 1. Also the procedure was similar. A slider was again used for the assessment of picture preference (from “I do not like it” to “I like it”). The task consisted of two blocks of 130 trials (all 130 stimuli). In each block the pictures were presented in a randomized order. The experiment lasted approximately 30 min.

### Results

The mean preference ratings ranged from 21.2 to 70.2 (*M* = 48.7, *SD* = 13.0). They were subjected to a within-participant one-way ANOVA with factor *stimulus type* (circles, or hexagons). The analysis revealed a significant difference, *F*_(1, 20)_ = 6.25, *p* < 0.05, $\eta_{p}^{2}=0.238$, indicating that pictures with circles were liked more than those with hexagons (52.8 vs. 44.6).

**Figure 2:**
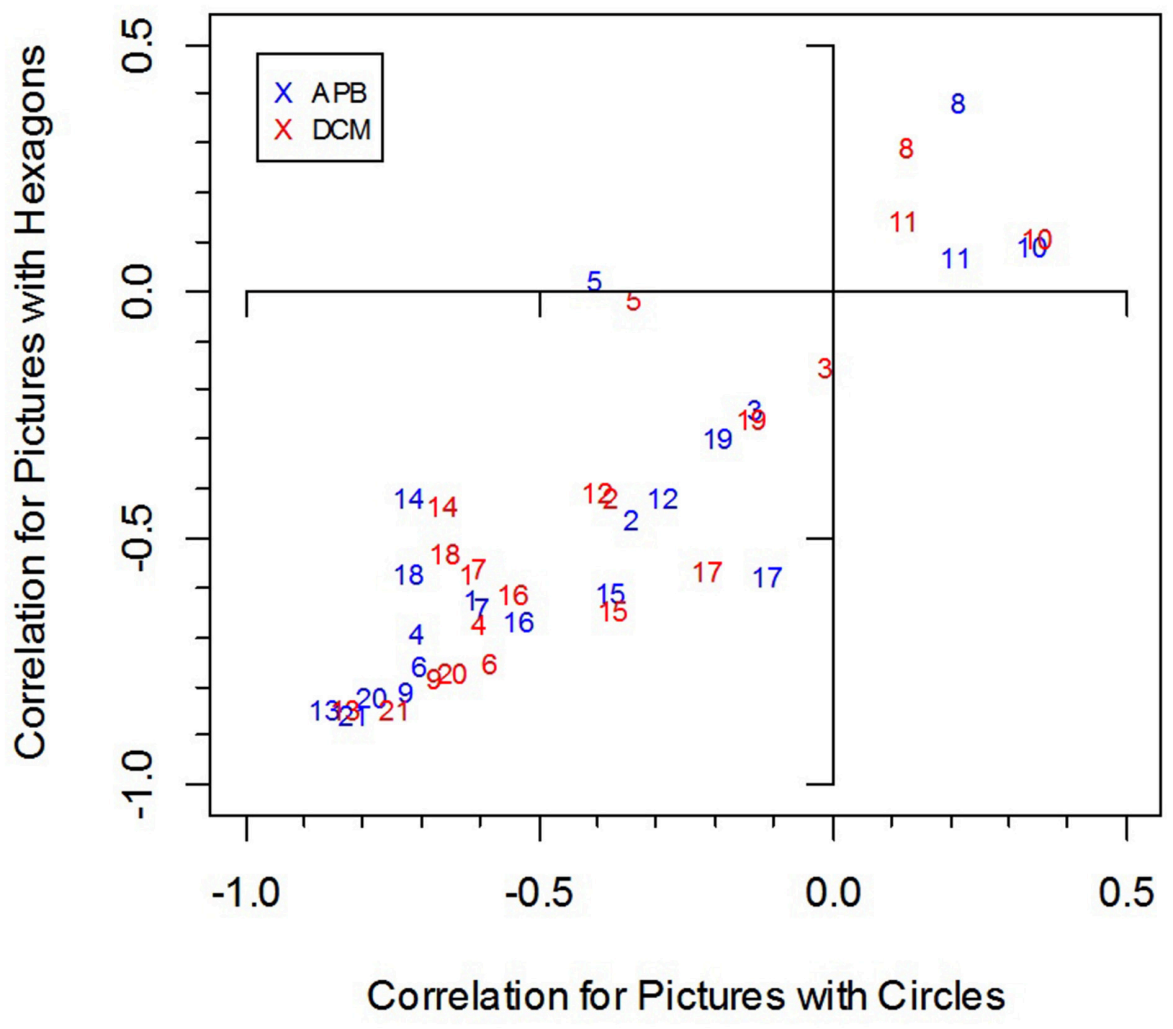
**Individual correlations between liking and the two measures *APB* and *DCM* for the two picture types (pictures with circles and with hexagons)**. The numbers indicate individual participants.

We computed for each participant the correlation between the preference ratings and the objective measures (*APB, DCM, MS*, and *HG)*. The mean correlations are shown in Table 7. As can be seen, they were substantial, except for the *MS* measure. However, the variability across participants was large (see Figure 2). The correlations between *APB* scores and liking ranged from −0.863 to 0.339 for the pictures with circles, and from −0.851 to 0.387 for the pictures with hexagons. Altogether, there were 5 participants with at least one non−significant (*p* > 0.05), correlation. Three participants produced at least one significant positive correlation. The correlations between *DCM* scores and liking ranged from −0.829 to 0.350 for the pictures with circles, and from −0.842 to 0.152 for the pictures with hexagons. There were 6 participants with at least one non-significant (*p* > 0.05) correlation, and two participants produced at least one significant positive correlation. The correlations were somewhat higher for the *APB* score than for the *DCM* score. A comparison across both stimulus types revealed a significant difference (−0.441 vs. −0.420), *F*_(1, 20)_ = 4.59, *p* < 0.05, $\eta_{p}^{2}=0.187$.

**Table 7:** **Means (across participants) of the individual correlations (across pictures) between the preference ratings and the different scores in Experiment 2**.

	***APB***	***DCM***	***MS***	***HG***
Liking Circles	−0.423 (0.363)	−0.401 (0.327)	0.084 (0.089)	0.408 (0.359)
Liking Hexagons	−0.459 (0.351)	−0.438 (0.340)	0.127 (0.110)	0.441 (0.320)

*The values in parenthesis are the standard deviations*.

*APB, Assessment of preference for balance; DCM, Deviation of the center of mass; MS, Mirror symmetry; HG, Homogeneity*.

The correlations between *HG* scores and liking ranged from −0.353 to 0.829 for the pictures with circles, and from −0.435 to 0.785 for the pictures with hexagons. Altogether, there were 5 participants with at least one non-significant (*p* > 0.05) correlation, and three participants produced at least one significant negative correlation. The correlations with liking were somewhat lower for the *HG* score than for the *APB* score, but higher relative to the *DCM* score. A comparison between the correlations for the *APB* and the *HG* scores across both stimulus types revealed no significant difference (−0.441 vs. −0.425), *F*_(1, 20)_ = 1.41, *p* = 0.249, $\eta_{p}^{2}=0.066$.

**Figure 3:**
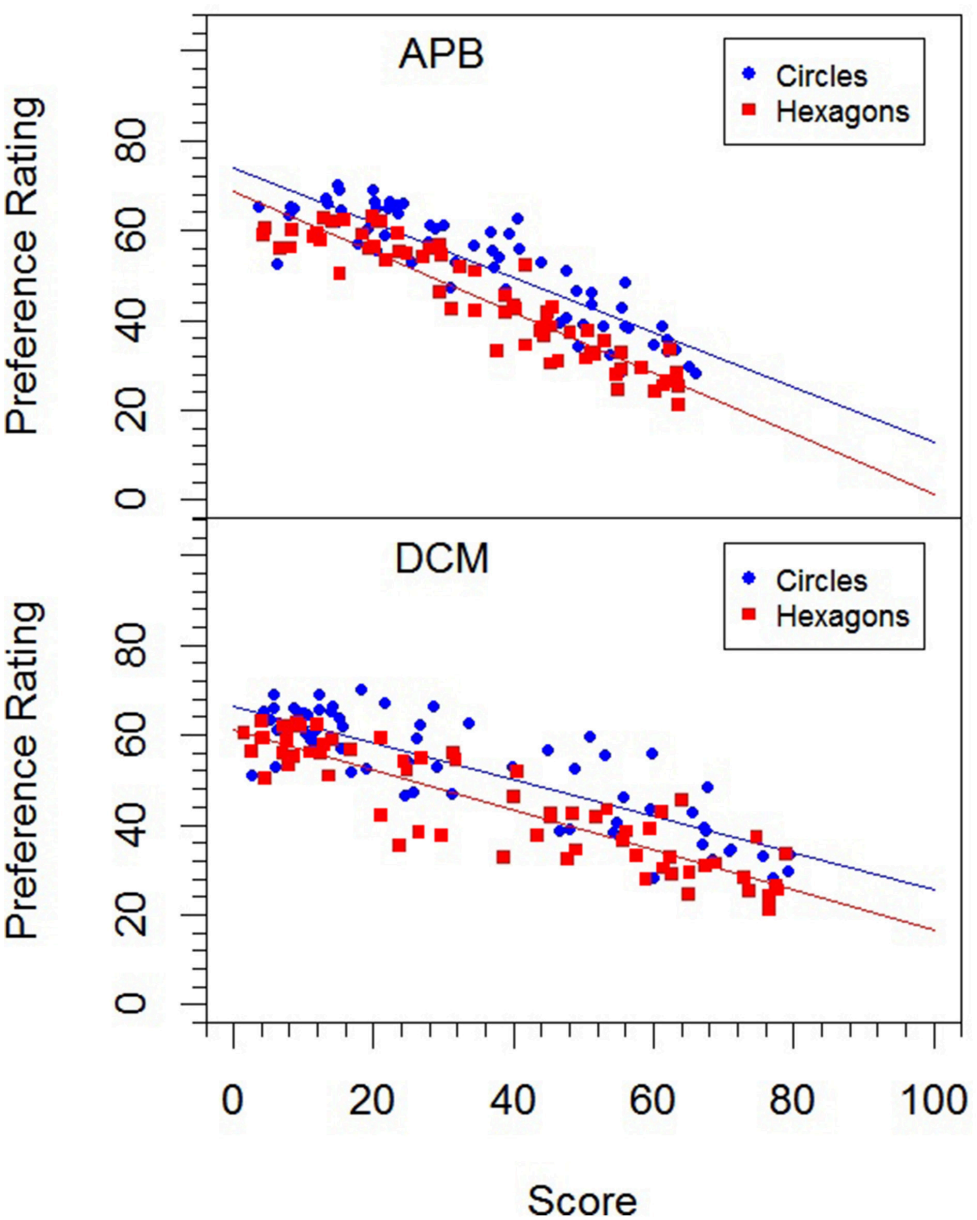
**Relation between preference ratings in Experiment 2 for the two picture types (circles, and hexagons) and the *APB* scores and *DCM* scores**.

The correlations of the mean preference ratings with the different measures and previous ratings are shown in Table 8. Obviously, the correlations with the ratings from Experiment 1 were rather high. Interestingly, liking correlated higher with symmetry than with balance. The correlations with the objective measures were also high, except for *MS*. The relation of the mean preference ratings with the *APB* scores and that with the *DCM* scores are also illustrated in Figure 3.

**Table 8:** **Correlations between the mean preference rating in Experiment 2 and the different scores and ratings from Experiment 1 across both stimulus types**.

		**Bal**.	**Sym**.	***APB***	***DCM***	***MS***	***HG***
Liking	All (*R*^2^)	0.816 (0.666)	0.900 (0.810)	−0.867 (0.752)	−0.844 (0.713)	0.199 (0.039)	0.848 (0.719)
	Circles (*R^2^*)	0.793 (0.629)	0.898 (0.807)	−0.882 (0.778)	−0.836 (0.699)	0.171^n.s.^ (0.029)	0.843 (0.710)
	Hexagons (*R*^2^)	0.843 (0.711)	0.930 (0.865)	−0.928 (0.862)	−0.892 (0.796)	0.256 (0.066)	0.875 (0.766)

*The values in parenthesis are the corresponding R^2^-values*.

n.s. not significant;

*Balance, Bal. rating (Experiment 1); Sym., Symmetry Rating (Experiment 1); APB, Assessment of preference for balance; DCM, Deviation of the center of mass; MS, Mirror symmetry; HG, Homogeneity*.

For the *APB* measure we also computed a multiple linear regression for analyzing to what extent its components were related to the preference ratings. The result is shown in Table 9. As can be seen, the explained variance across both stimulus types increased only slightly from 75.2 to 78.5%, compared to the original scores. Interestingly, the inner-outer components had small but reliable effects.

**Table 9:** **Regressions of preference ratings from Experiment 2 on the components of the APB score**.

	**Circles (*****R***^**2**^ = 0.809**)** ***F***_**(8, 56)**_ = 29.6, ***p*** < 0.001 **Intercept** = **726.1**		**Hexagons (*****R***^**2**^ = 0.885**)** ***F***_**(8, 56)**_ = 53.6, ***p*** < 0.001 **Intercept** = **67.0**		**Both (*****R***^**2**^ = 0.785**)** ***F***_**(8, 121)**_ = 55.2, ***p*** < 0.001 **Intercept** = **69.8**	
	**β**	***P(>|t|)***	**β**	***P(>|t|)***	**β**	***P(>|t|)***
*h*	−0.086	0.036^*^	−0.101	0.000^***^	−0.082	0.003^**^
*v*	−0.038	0.204	−0.101	0.001^***^	−0.074	0.003^**^
*ad*	−0.109	0.005^**^	−0.094	0.002^**^	−0.113	0.000^***^
*md*	−0.115	0.000^***^	−0.097	0.001^**^	−0.121	0.000^***^
*h_*io*_*	−0.063	0.096	−0.062	0.038^*^	−0.050	0.067
*v_*io*_*	−0.026	0.440	−0.034	0.230	−0.024	0.358
*ad_*io*_*	−0.048	0.165	−0.071	0.017^*^	−0.066	0.013^*^
*md_*io*_*	−0.078	0.041^*^	−0.037	0.183	−0.049	0.066

* p < 0.05;

** p < 0.01;

*** *p < 0.001*.

### Discussion

In this experiment the participants had to judge how much they liked the pictures from the APB also applied in Experiment 1. First of all, pictures with circles were liked more than those with hexagons, which supports the hypothesis of Silvia and Barona (2009) that curved objects are preferred to angular ones. If we consider the relation between liking and the judgments from Experiment 1, then liking correlated higher with the pictures' rated symmetry than with their rated balance. This suggests that aesthetic preference is more affected by symmetry perception than by balance perception. However, it remains somewhat unclear how the participants operationalized these two concepts.

With respect to the *APB* measure, we replicated Wilson and Chatterjee's (2005) result. The scores correlated substantially with the mean preference ratings (see Figure 3). This seems to contradict Silvia and Barona's non-significant results. However, these researchers used only a small selection of 18 pictures from the APB and analyzed individual correlations rather than the correlation between the average ratings and *APB* scores. As we have seen, individual correlations can be much lower and vary considerably across participants (see Figure 2).

The *DCM* scores also correlated highly with the preference ratings, although significantly less than the *APB* scores. The smallest correlation occurred between the *MS* scores and the preference ratings.

A multiple linear regression of the preference ratings on the different components of the *APB* score revealed a reliable effect of inner-outer components (see Table 9). Because these components are related to homogeneity, there was also a corresponding high correlation between liking and the measure *HG*, which was not significantly different from that between liking and *APB* scores.

## Experiment 3

In our third experiment we wanted to see how general the obtained results are, i.e., to what extent they depended on the specific stimulus set. Wilson and Chatterjee's (2005) constructed their stimuli manually with a drawing program in such a way that their sets covered a large range of *APB* scores. This construction process might have produced systematic relations between stimulus features which are favorable for the correlation of *APB* scores with ratings of balance and liking. For instance, if we consider Figure 3, then we see that the *APB* scores of the pictures are indeed rather evenly distributed whereas the *DCM* scores cluster somewhat at smaller values. That is, the center of “mass” of many APB stimuli is close to the geometric center. To additionally apply stimuli whose *DCM* scores are more evenly distributed, we constructed new pictures which also contained seven circles or seven hexagons of different size (examples are shown in the right panel of Figure 1). However, the positions of the elements in each picture were randomly drawn from a two-dimensional Gaussian distributions with specific mean and variance. For the new pictures the *APB* and *DCM* scores were somewhat smaller, compared to the APB stimuli. Homogeneity and mirror symmetry were also reduced.

Different from Wilson and Chatterjee's (2005), we used the same positions for constructing pictures with circles and for those with hexagons, which allowed us a more reliable comparison between the ratings for these stimulus types. Finally, we required preference as well as balance ratings from the same participants.

### Method

Twenty-seven students from the University of Konstanz were recruited via an online recruitment system (ORSEE, Greiner, 2015) for participating in the experiment. The data of four participants were excluded from data analysis, because their balance ratings were opposite to those of the other participants, which indicates that they did not understand the task correctly. The remaining 23 participants (4 males) had an average age of 22 years (*SD* = 3.18). All had normal or corrected-to-normal vision and were paid 5 € for their participation. The experiment was performed under the same ethical standards as the previous experiments.

As stimuli served new sets of pictures. Each picture had an extension of 500 × 500 pixels, which approximately corresponded to a visual angle of 14° horizontally and vertically. As elements served either seven circles or seven hexagons of different size. Examples are shown in the right panel of Figure 1. As can be seen, the elements were somewhat smaller than those in the APB stimuli. The location of the elements was established by a random process. The positions for each picture were drawn from a two-dimensional Gaussian distribution under the restriction that the elements do not overlap. The mean of the distribution could be one of the nine combinations resulting from three horizontal (left, center, and right) and three vertical (top, center, and bottom) positions. Most pictures (53) were constructed from a distribution with a “center-center” mean, i.e., a mean that corresponded to the geometric center. To construct less balanced pictures, also the other means, e.g., “top-left” were used. Additionally, the variances were reduced. A set of 72 pictures for each element type (circles, hexagons) was put together such that *DCM* scores were evenly distributed. For the new stimulus sets the *APB* scores ranged from 23.9 to 72.4 (*M* = 49.0, *SD* = 12.3), and the *DCM* scores from 4.30 to 83.0 (*M* = 44.7, *SD* = 25.3). Homogeneity (entropy) ranged from 58.4 to 85.5 (*M* = 70.3, *SD* = 5.48), and mirror symmetry from 0.29 to 4.02 (*M* = 1.22, *SD* = 0.616).

The experimental procedure was similar to that in our previous experiments, except that participants rated each picture with respect to balance and liking. There were two blocks of 144 trials (all 144 stimuli) each. In each block the pictures were presented in a randomized order. In the first block the participants had to judge how much the liked each picture, and in the second block they had to rate the pictures' balance. The experiment lasted approximately 30 min.

### Results

We first computed the mean balance ratings and the mean preference ratings across participants for each picture and correlated the obtained values across all 144 pictures with the different scores. The resulting correlations and corresponding *R*^2^-values are shown in Table 10. Moreover, the correlations between the different measures are also shown in this table. As can be seen, compared to the previous experiments, the correlations between the mean *APB* scores and the other measures were generally smaller. The same holds for the *DCM* measure, except for the correlation with *MS*. The relations between the ratings and the *APB* scores and *DCM* scores were further analyzed.

**Table 10:** **Correlations between the mean ratings in Experiment 3 and the different scores across both stimulus types**.

	**Liking**	**Balance**	***APB***	***DCM***	***MS***	***HG***
Liking	–	0.865^***^	−0.737^***^	−0.742^***^	0.296^***^	0.697^***^
Balance	0.748	–	−0.836^***^	−0.916^***^	0.364^***^	0.661^***^
*APB*	0.544	0.699^***^	–	0.793^***^	−0.139^**^	−0.689^***^
*DCM*	0.552	0.838^***^	0.628^***^	–	−0.454^***^	−0.558^***^
*MS*	0.088	0.132^***^	0.019^***^	0.206	–	0.194^**^
*HG*	0.486	0.437^***^	0.474^***^	0.311	0.003	–

*The values below the diagonal are the corresponding R^2^-values*.

** p < 0.01;

*** *p < 0.001*.

*APB, Assessment of preference for balance; DCM, Deviation of the center of “mass”; MS, Mirror symmetry; HG, Homogeneity*.

#### Balance ratings

The mean balance ratings, which ranged from 8.09 to 69.3 (*M* = 42.2, *SD* = 16.4), were subjected to a within-participant ANOVA with factor *stimulus type* (circles, or hexagons). The analysis revealed no significant difference, $F_{\left(1,\;23\right)}=0.02,p=0.56,\eta_{p}^{2}=0.015$. Mean balance for pictures with circles and for those with hexagons were almost identical (circles: 42.4, hexagons: 42.0). Next, we computed for each participant the correlation between the balance ratings and the relevant scores across the 72 pictures with circles and across the 72 pictures with hexagons.

##### APB

For the pictures with circles, the correlations between the *APB* scores and the balance ratings ranged from −0.818 to −0.187, and for those with hexagons from −0.658 to −0.146. There was only one participant with non-significant (*p* > 0.05) correlations for both stimulus types. Three participants had a non-significant correlation for one of the stimulus types. The mean correlations for the circles and hexagons were −0.601 (*SD* = 0.169) and −0.495 (*SD* = 0.147), respectively.

##### DCM

Correlations between the *DCM* scores and the balance ratings ranged from −0.853 to −0.258 for pictures with circles, and from −0.775 to −0.146 for pictures with hexagons. There was no participant with non-significant (*p* > 0.05) correlations for both stimulus types. Two participants had a non-significant correlation for one of the stimulus types. The mean correlations for the circles and hexagons were −0.669 (*SD* = 0.172) and −0.581 (*SD* = 0.176), respectively.

##### HG

Correlations between the *HG* scores and the ratings ranged from 0.128 to 0.756 for pictures with circles, and from 0.169 to 0.596 for pictures with hexagons. One participants had non-significant (*p* > 0.05) correlations for both stimulus types, and two participants had a non-significant correlation for one of the stimulus types. The mean correlations for the circles and hexagons were 0.514 (*SD* = 0.161) and 0.398 (*SD* = 0.134), respectively.

The correlation values (for HG the values were multiplied by −1) were entered to an ANOVA with the two within-participant factors *score* (*APB, DCM*, or *HG*), and *stimulus type* (circles, or hexagons). It revealed significant main effects. The correlations between the scores and ratings were significantly higher (−0.597 vs. −0.491) for pictures with circles than for those with hexagons, *F*_(1, 22)_ = 65.8, *p* < 0.001, $\eta_{p}^{2}$ = 0.749. Moreover, the correlations differed reliably between the scores *F*_(2, 44)_ = 27.9, *p* < 0.001, $\eta_{p}^{2}$ = 0.559. Further analyses showed that the correlations were significantly higher for the *DCM* scores than for the *APB* scores (−0.625 vs. −0.551), *F*_(1, 22)_ = 13.2, *p* < 0.01, $\eta_{p}^{2}$ = 0.376, and for *APB* scores than for the *HG* scores (−0.551 vs. −0.456), *F*_(1, 22)_ = 42.5, *p* < 0.001, $\eta_{p}^{2}=0.659$. These relations also corresponds to the correlations with the mean ratings (see Table 10).

#### Preference ratings

**Figure 4:**
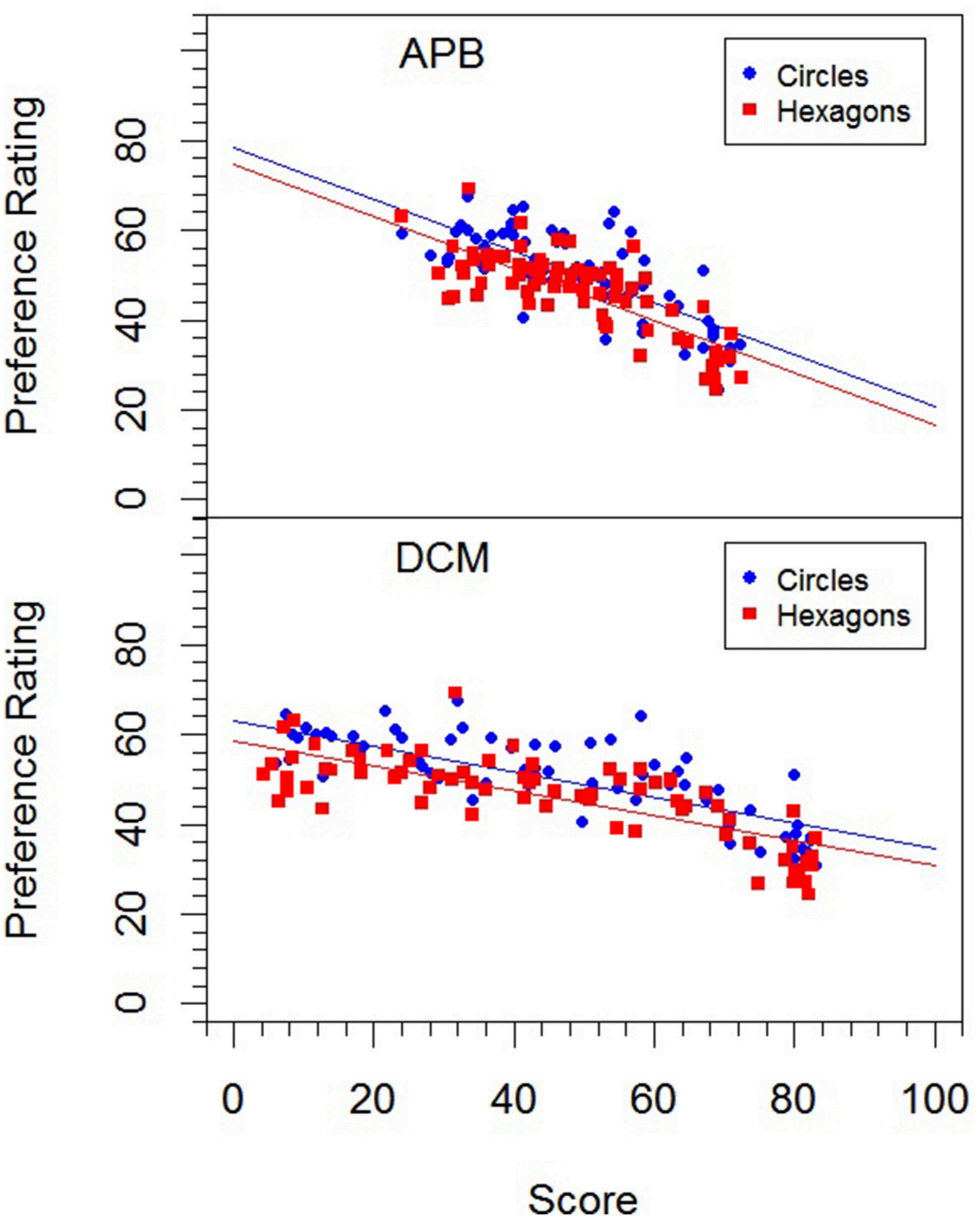
**Relation between preference ratings in Experiment 3 for the two picture types (circles, and hexagons) and the *APB* scores and *DCM* scores**.

The mean preference ratings ranged from 24.3 to 69.2 (*M* = 48.2, *SD* = 9.61). They were subjected to a within-participant one-way ANOVA with factor *stimulus type* (circles, or hexagons). The analysis revealed a significant difference, *F*_(1, 22)_ = 7.75, *p* < 0.05, $\eta_{p}^{2}$ = 0.261, indicating that pictures with circles were liked more than those with hexagons (50.3 vs. 46.2). This difference can also be seen in Figure 4, where the relations between the mean preference ratings and the *APB* scores and *DCM* scores are shown by scatterplots.

##### APB

The average of the correlations between the *APB* scores and the preference ratings was −0.276. For the pictures with circles the correlations ranged from −0.715 to 0.419, and for those with hexagons from −0.658 to −0.534. Four participants had non-significant (*p* > 0.05) correlations for both stimulus types. Three participants had a non-significant correlation for one of the stimulus types. The mean correlations for the circles and hexagons were −0.310 (*SD* = 0.356) and −0.241 (*SD* = 0.330), respectively.

##### DCM

The average of the correlations between the *APB* scores and the preference ratings was −0.292. The correlation ranged from −0.706 to 0.391 for pictures with circles, and for those with hexagons from −0.744 to 0.564. Four participants had non-significant (*p* > 0.05) correlations for both stimulus types, and four participants had a non-significant correlation for one of the stimulus types. The mean correlations for the circles and hexagons were −0.315 (*SD* = 0.318) and −0.269 (*SD* = 0.316), respectively.

##### HG

The average of the correlations between the *HG* scores and the preference ratings was −0.265. The correlation between the *HG* measures and the pictures with circles ranged from −0.484 to 0.642, and for those with hexagons from −0.467 to 0.552. Five participants had non-significant (*p* > 0.05) correlations for both stimulus types, and four participants had a non-significant correlation for one of the stimulus types. The mean correlations for the circles and hexagons were 0.310 (*SD* = 0.345) and 0.220 (*SD* = 0.282), respectively.

The correlation values (for HG the values were multiplied by −1) were entered to an ANOVA with the two within-participant factors *score* (*APB, DCM*, or *HG*), and *stimulus type* (circles, or hexagons). It revealed a significant main effect of *stimulus type*. The correlations between the scores and the preference ratings were significantly higher (-0.312 vs. −0.243) for pictures with circles than for those with hexagons, *F*_(1, 22)_ = 5.26, *p* < 0.05, $\eta_{p}^{2}=0.193$. The factor *score* (*DCM* = −0.292, *APB* = −0.276, *HG* = −0.265) was not significant, *F*_(2, 44)_ = 1.39, *p* = 0.260, $\eta_{p}^{2}=0.059$.

### Discussion

In this experiment we applied new sets of pictures with circles or hexagons, whose element positions were selected by a random process. On average, the pictures were less balanced, mirror symmetric, and homogenous, compared to the APB stimuli. The reduced objective balance is also reflected by the somewhat smaller balance ratings, compared to Experiment 1. If we consider the different measures, then their correlations with the balance ratings were significantly higher for the *DCM* scores than for the *APB* scores, and those for the *APB* scores were significantly higher than those for the *HG* scores. This relation also holds for the correlation with the mean balance ratings (see Table 10). These results demonstrate again that the *DCM* measure is well-suited for assessing the balance of simple pictures. However, our results also show that the correlations depended on the form of the picture elements. They were significantly higher for pictures with circles than for those with hexagons. We can take this result seriously, because in our stimuli circles and hexagons had identical positions in the corresponding pictures.

In contrast to the balance ratings, the range and mean of the preference ratings for the new pictures are similar to those in Experiment 2. Moreover, pictures with circles were again preferred to those with hexagons. This effect was even more pronounced than in Experiment 2, presumably because our picture types had identical element locations.

With respect to the correlations of the preference ratings with the different measures, we found no reliable difference between the *APB, DCM*, and *BH* scores. However, stimulus type again modulated the correlations. For pictures with circles they were reliably higher than for those with hexagons.

Taken together, our results with the new stimuli are similar to those obtained with the APB stimuli in the previous two experiments. However, the advantage of the *DCM* scores as measure of balance, compared to the *APB* scores, came out more clearly. Moreover, the advantage of the *APB* scores over the *DCM* scores for predicting liking vanished. This demonstrates that the extent to which the different measures account for balance and preference ratings, depends on the specific selection of pictures, even if the pictures are rather similar.

## General discussion

There is a wide agreement that pictorial balance is crucial for the aesthetic appreciation of pictures (e.g., Poore, 1903; Arnheim, 1954; Locher et al., 1996). However, the mechanisms of balance perception and their relation to aesthetics are still not well understood. A promising approach to shed some light onto these mechanisms has been to create objective measures of balance that correlate with aesthetic preference. Therefore, the aim of the present study was to compare currently applied measures by examining how well they account for balance, symmetry, and preference judgments of simple pictures.

One of the considered measures of balance, the *DCM*, is based on the theory of Arnheim (1954) and his precursors (e.g., Poore, 1903; Ross, 1907). The idea of these researchers was that each picture has a center of perceptual “mass,” and that the more this center deviates from the picture's geometric center, the less balanced the picture is. Although it has been shown that such a measure does not generally correlate with aesthetic preference (e.g., McManus et al., 2011), we hypothesized that it might work for the simple pictures used in this study.

Another measure that was less inspired by a physical metaphor was the *APB* score (Wilson and Chatterjee, 2005). It is defined by the mean of eight partial measures that are more or less related to symmetry. For comparison, we also included a more strict measure of symmetry. Specifically, we computed a score *MS* that represents the degree of mirror symmetry around four axes. Finally, we applied a measure *HG* that reflects the homogeneity of the elements in a picture.

In our first experiment, participants judged the perceptual balance and symmetry of simple pictures taken from Wilson and Chatterjee's (2005) APB test. The pictures consisted of seven circles or hexagons of different size. Our results show that, although there was some variance across participants, the *DCM* and *APB* scores correlated rather high with the balance and symmetry mean ratings. The high correlation of the *APB* scores with the balance ratings replicates the result of Wilson and Chatterjee's (2005). For examining to what extent the individual components of this measure were related to the balance ratings, we entered the components into a multiple linear regression. The analyses revealed that the horizontal dimension had the largest weight, followed by the vertical dimension. The other components had little or no effect. This result suggests that a differential weighting of the components can improve the predictive power of the *APB* scores with respect to balance ratings. Such a modification of the original measure would also remedy one of its deficits. As has been shown, balance perception strongly depends on the orientation of a picture (Gershoni and Hochstein, 2011). The *APB* measure, however, is invariant with respect to image rotation.

Interestingly, the *DCM* scores correlated numerically higher with the balance and symmetry ratings than the *APB* scores. This demonstrates that the traditional idea of a center of perceptual “mass” is an adequate account of perceptual balance, at least for simple pictures. Inventing a new score would not have been necessary. Moreover, a detailed analysis revealed that the *APB* measure is inconsistent to some extent. Pictures whose elements are only located in the central area receive a relatively high score (poor balance), although they were rated as highly balanced. Responsible for this inconsistency are the inner-outer components of the *APB* measure, which reflect to a large degree homogeneity. In the present case the effects of the inconsistency remained relatively weak, because there were only few pictures of this type.

Obviously, only if the center of “mass” is near the geometric center, homogeneity can vary freely from minimum to maximum. The closer the center of “mass” moves to the border, i.e., the less balanced a picture, the more restricted homogeneity. That is, in the less balanced pictures the elements are less scattered. Consequently, balance and homogeneity are correlated across stimuli. This confound was responsible for the correlation between the homogeneity scores and the balance ratings. However, the correlation was smaller than those for the *DCM* and *APB* scores. The smallest correlations were found for the *MS* scores. This suggests that the visual system is not very sensitive to mirror symmetry, at least if the elements are scattered as in the present study.

To see how the ratings and measures are related to aesthetic preference ratings, we conducted a second experiment in which (different) participants had to rate how much they liked the APB pictures. First of all, we found that the pictures with circles were liked more than those with hexagons. This replicates results from Silvia and Barona (2009) and supports the hypothesis that pictures with curved elements are preferred to those with angular elements (Bar and Neta, 2006). Furthermore, correlating the preference ratings with the ratings from Experiment 1 revealed that liking correlated higher with symmetry than with balance. However, this result is not easy to interpret, because symmetry and balance ratings were highly correlated. Moreover, rating the balance of pictures was presumably more difficult to conceptualize than rating their symmetry.

The correlations between the preference ratings and the different measures were generally rather high except for the *MS* scores. However, the correlations with the *APB* scores were significantly higher than those with the *DCM* scores. Interestingly, the correlation with the *HG* scores did not differ significantly from those with the *APB* scores. This indicates that homogeneity, in addition to balance, also affected preference, which, in turn, explains why the *APB* scores correlated higher with liking than the *DCM* scores. For predicting linking, the inner-outer components of the *APB* measure, which largely reflect homogeneity, came favorably into play.

Thus, the first two experiments show that the *DCM* scores can account similarly well as the *APB* scores for the balance ratings, but might be preferred, because the latter measure can lead to inconsistencies. For predicting preference, the *APB* scores were superior to the *DCM* scores, mainly because they take homogeneity into account. That homogeneity is a crucial feature is supported by the fact that the *HG* scores also accounted well for the preferences and, therefore, could be used alternatively. As one reviewer pointed out, the fact that homogeneity played an important role for preference is compatible with Arnheim's (1954) idea that also the corners of a frame exert some “force” on the elements.

Because these results in our first two experiments were obtained with a specific selection of stimuli that were presumably constructed to be optimal for the *APB* scores, we wanted to examine to what extent the results generalize to a different set of stimuli. For this objective we conducted a third experiment with new pictures that were also constructed from circles and hexagons, but whose element locations were drawn randomly from a two-dimensional Gaussian distribution. The resulting pictures are less balanced than those from the APB, but the corresponding *DCM* scores are more evenly distributed. Moreover, the correlations between the different measures are reduced, except for mirror symmetry. The participants in Experiment 3 had to rate both balance and preference. Whereas the obtained balance ratings where somewhat smaller than those in Experiment 1, the preference ratings were similar to those in Experiment 2. Pictures with circles were again preferred to those with hexagons. Moreover, the correlation between the balance ratings and the *DCM* scores was significantly higher than that with the *APB* scores, whereas the correlations with the preference ratings did not significantly differ between *DCM, APB*, and *HG* scores. These results demonstrate that the performance of the different scores depend, at least to some extent, on the specific selection of pictures, even if they look rather similar.

Taken together, our experiments and analyses demonstrate that the *APB* score is not a pure measure of balance. Therefore, if one is interested in predicting perceptual balance, then the *DCM* measure is the better choice, mainly because it is less affected by homogeneity. If the goal is to predict preference ratings for pictures, then the *APB* score is appropriate. Our results indicate that preference not only depends on balance, but also on homogeneity, which is taken into account by the *APB* measure. However, *APB* scores can be substituted by *HG* scores, which produced comparable results. Their advantage is that they are rather simple to compute and easy to comprehend.

Further studies will have to show to what extent the considered measures can also predict aesthetic preferences for more complex pictures, e.g., for those also including objects. A related question is whether the present ratings were the result of a global impression formed during the “first glance” (Locher, 2015), or also of a deeper processing. The registration of eye movements during the rating period would presumably be helpful in this respect. Finally, it should be noted that we considered a selection of proposed or possible objective measures for predicting perceptual balance and aesthetic preference. Therefore, it would be interesting to compare other scores as well. For instance, for the type of pictures applied in this study measures reflecting the goodness of dot patterns (e.g., Van Der Helm and Leeuwenberg, 1996) are promising candidates.

## Author contributions

RH: conception, design, final approval, accountable for quality. MF: conception, data aquisition, drafting, revising, interpretation.

### Conflict of interest statement

The authors declare that the research was conducted in the absence of any commercial or financial relationships that could be construed as a potential conflict of interest.

## References

[B1] Arnheim R. (1954). Art and Visual Perception: A Psychology of the Creative Eye. Berkeley, CA; Los Angeles, CA: University of California Press.

[B2] Bar M. Neta M. (2006). Humans prefer curved visual objects. Psychol. Sci. 17, 645–648. 10.1111/j.1467-9280.2006.01759.x 16913943

[B3] Bauerly M. Liu Y. (2006). Computational modeling and experimental investigation of effects of compositional elements on interface and design aesthetics. Int. J. Hum. Comput. Stud. 64, 670–682. 10.1016/j.ijhcs.2006.01.002

[B4] Cupchik G. C. (2007). A critical reflection on Arnheim's Gestalt theory of aesthetics. Psychol. Aesthet. Creat. Arts 1:16 10.1037/1931-3896.1.1.16

[B5] Fechner G. T. (1871). Zur experimentalen Ästhetik. Abh. der Königlich Sächsischen Ges. der Wissenschaften 9, 555–635. 19552825

[B6] Fechner G. T. (1876). Vorschule der Ästhetik. Leipzig: Breitkopf and Härtel.

[B7] Garner W. R. Clement D. E. (1963). Goodness of pattern and pattern uncertainty. J. Verbal Learn. Verbal Behav. 2, 446–452.

[B8] Gershoni S. Hochstein S. (2011). Measuring pictorial balance perception at first glance using Japanese calligraphy. i-Perception 2, 508. 10.1068/i0472aap 23145242 PMC3485800

[B9] Greiner B. (2015). Subject pool recruitment procedures: organizing experiments with ORSEE. J. Econ. Sci. Assoc. 1, 114–125. 10.1007/s40881-015-0004-4

[B10] Jacobsen T. Höfel L. (2002). Aesthetic judgments of novel graphic patterns: analyses of individual judgments. Percept. Mot. Skills 95, 755–766. 10.2466/pms.2002.95.3.755 12509172

[B11] Latto R. (1995). The brain of the beholder, in The Artful Eye, eds Gregory L. R. Harris J. J. Heard P. Rose D. (Oxford: Oxford University Press), 66–94.

[B12] Locher P. (2015). The aesthetic experience with visual art ‘at first glance’, in Investigations into the Phenomenology and the Ontology of the Work of Art, Vol. 81, eds Bundgaard P. F. Stjernfelt F. (New York, NY: Springer International Publishing), 75–88.

[B13] Locher P. Gray S. Nodine C. (1996). The structural framework of pictorial balance. Perception 25, 1419–1436. 10.1068/p251419

[B14] Locher P. Nodine C. (1989). The perceptual value of symmetry. Comput. Math. Appl. 17, 475–484. 10.1016/0898-1221(89)90246-0

[B15] McManus I. Edmondson D. Rodger J. (1985). Balance in pictures. Br. J. Psychol. 76, 311–324. 10.1111/j.2044-8295.1985.tb01955.x

[B16] McManus I. Stöver K. Kim D. (2011). Arnheim's Gestalt theory of visual balance: examining the compositional structure of art photographs and abstract images. i-Perception 2, 615. 10.1068/i0445aap 23145250 PMC3485801

[B17] Munar E. Gómez-Puerto G. López-Navarro E. Nadal M. (2014). Visual preference for curvature as a potential aesthetic primitive, in Proceedings of the Twenty-Third Biennial Congress of the International Association of Empirical Aesthetics, ed Kotzbelt A. (New York, NY), 316–319.

[B18] Ngo D. C. L. Teo L. S. Byrne J. G. (2002). Evaluating interface esthetics. Knowl. Inf. Syst. 4, 46–79. 10.1007/s10115-002-8193-6

[B19] Palmer S. E. Schloss K. B. Sammartino J. (2013). Visual aesthetics and human preference. Annu. Rev. Psychol. 64, 77–107. 10.1146/annurev-psych-120710-100504 23020642

[B20] Poore H. R. (1903). Pictorial Composition and the Critical Judgement of Pictures. New York, NY: Baker and Taylor.

[B21] Ross D. W. (1907). A Theory of Pure Design: Harmony, Balance, Rhythm. Boston, MA: Houghton, Mifflin.

[B22] Shannon C. E. (1948). A mathematical theory of communication. Bell Syst. Tech. J. 27, 379–423, 623–656. 10.1002/j.1538-7305.1948.tb00917.x

[B23] Silvia P. J. Barona C. M. (2009). Do people prefer curved objects? Angularity, expertise, and aesthetic preference. Empir. Stud. Arts 27, 25–42. 10.2190/EM.27.1.b

[B24] Treiblmaier H. Filzmoser P. (2009). Benefits from Using Continuous Rating Scales in Online Survey Research. Vienna: University of Technology, Research Report SM-2009–M-2004.

[B25] Van Der Helm P. A. Leeuwenberg E. L. (1996). Goodness of visual regularities: a nontransformational approach. Psychol. Rev. 103:429. 10.1037/0033-295X.103.3.429 8759043

[B26] Wilson A. Chatterjee A. (2005). The assessment of preference for balance: introducing a new test. Empir. Stud. Arts 23, 165–180. 10.2190/B1LR-MVF3-F36X-XR64

